# Influence of Immediate Implant Placement and Provisionalization with or without Soft Tissue Augmentation on Hard and Soft Tissues in the Esthetic Zone: A One-Year Retrospective Study

**DOI:** 10.1155/2021/8822804

**Published:** 2021-01-05

**Authors:** Paolo De Angelis, Paolo Francesco Manicone, Giulio Gasparini, Silvio De Angelis, Margherita Giorgia Liguori, Ilaria De Filippis, Antonio D'Addona

**Affiliations:** ^1^Department of Head and Neck, Division of Oral Surgery and Implantology, Institute of Clinical Dentistry, Fondazione Policlinico Universitario A. Gemelli IRCCS, Università Cattolica del Sacro Cuore, Rome, Italy; ^2^Department of Head and Neck, Division of Oral and Maxillofacial Surgery, Institute of Clinical Dentistry, Fondazione Policlinico Universitario A. Gemelli IRCCS, Università Cattolica del Sacro Cuore, Rome, Italy; ^3^Private Dental Practice, Ascoli Piceno, Italy

## Abstract

The purpose of this clinical research was to evaluate peri-implant marginal changes around immediate implants placed either with the application of SCTG or XCM or without soft tissue grafting. A total of 48 patients requiring a single implant-supported restoration in the anterior jaw were selected for inclusion. Three surgical procedures were performed, as follows: type 1 implant with subepithelial connective tissue graft (SCTG), type 1 implant with xenogenic collagen matrix (XCM), and type 1 implant without soft tissue augmentation (NG) (control group). The marginal change of peri-implant soft tissue, facial soft tissue thickness (FSTT), peri-implant health status, esthetics, and patient satisfaction were assessed at one year after surgery. All of the placed implants showed a survival rate of 100%. No significant differences in FSTT were recorded between the SCTG group and the XCM group after treatment (*P* > 0.05), while the NG group presented a significant difference (*P* < 0.05). Patients in the NG group lost significantly more in the buccal marginal level than did patients in the SCTG group and those in the XCM group (*P* < 0.05). The favourable success rate recorded in all groups confirmed immediate tooth replacement as a choice of treatment for a missing anterior single tooth. The NG group presented significant changes of FSTT and buccal marginal level, while XCM constituted a viable alternative to SCTG.

## 1. Introduction

After having a tooth taken out, biological occurrences happen while the extraction socket heals, causing marked changes in the hard and soft tissue volumes [1, 2]. Regarding esthetics, teeth replacement can be performed with implants, following various clinical protocols, when there is a necessary amount of bone and when there is a favourable volume of the alveolar ridge [3, 4].

In the literature, a lot of interest has taken place regarding type 1 implants, which are placed immediately after extraction and provisionalized within 24 hours (IIPP) [1, 4, 5]. Among the advantages of this approach exist the possibilities of lowering the amount of surgeries and the time required without affecting the predictability in terms of survival [1, 2, 4, 6]. However peri-implant tissue stability is a key factor that can impact the success of implant restorations because the postoperative tissue remodelling has the potential to compromise the esthetic results [7, 8]. Here, potential risk factors can be divided into intrinsic and extrinsic ones [9]. Prognostic factors are dependent on the thickness of the patient's buccal bone wall and soft tissue phenotype [9–11]. It has been clearly revealed that the buccal plate, especially in the coronal part, usually is composed solely of bundle bone, which is resorbed after the tooth extraction [12, 13]. Since the normal amount of the covering soft tissue varies from 2.8 mm to 3.8 mm, the bone resorption can cause various degrees of soft tissue shrinkage in the midfacial area [14, 15]. Furthermore, the loss of teeth changes the amount of soft tissue available and it is not clear whether bone resorption can be reduced by thickening the soft tissue [14].

IIPP does not avoid loss of the buccal bone wall nor mucosal recession or ridge dimensional changes [16, 17]. For this reason, different approaches, which were combined or used separately, have been introduced to minimize hard and soft tissue volume alterations and improve esthetic aspects using such methods as bone grafts and soft tissue grafting [16, 18, 19]. Extrinsic factors such as a proper placement of the implant and a suitable use of the provisional restoration are operator dependent. A key factor to improve the esthetics and reduce the negative aspects of bone resorption in the buccal area is an implant placement position of a minimum of 2 mm from the buccal wall of the alveolus and the filling of the space by using a bone graft [11]. In addition, it might be advantageous to place a slightly undersized implant to preserve more buccal soft and hard tissue volume [20].

The autogenous connective tissue graft (SCTG) is considered the optimum for building up soft tissue [18] and has been extensively used and investigated, demonstrating a clinical superiority when compared with xenogenic collagen matrix (XCM) [16]. XCM was introduced to overcome the disadvantages of autogenous soft tissue graft, which include increased patient morbidity and a reduced quantity of tissue available, and subsequently demonstrated positive short-term outcomes [18].

The advantages of soft tissue augmentation reported in the literature include better esthetics, the maintenance of soft tissue volume and marginal level, and a greater soft tissue thickness [16]. Furthermore, the characteristics of the soft tissue seem able to affect peri-implant status and greater soft tissue thickness can allow us to cover up the grey colour of the titanium [18, 21]. However, there is no agreement on the advantages of combining the immediate implant placement with soft tissue grafting, because successful outcomes can be obtained also without soft tissue grafting; furthermore, there is a low level of evidence on the use of xenogenic collagen matrix [16].

The purpose of this clinical research was to evaluate peri-implant marginal changes seen with immediate implants placed either with the application of SCTG or XCM or without soft tissue grafting.

## 2. Materials and Methods

Forty-eight patients from a private practice who needed a single implant-supported rehabilitation in the anterior upper or lower jaw (i.e., incisors, canine, or first premolars) were selected for inclusion in this one-year retrospective study; studies were conducted between 1 January 2017 and 1 January 2018.

The eligibility criteria stipulated that patients must have a general health showing no contraindications for surgery, over 20 years old, must have no signs of acute disease in the implant area, or signs of uncontrolled periodontal disease. As well, they must have both adjacent and opposing teeth and not require restorative treatment of them. They must also demonstrate sufficient mesiodistal and interocclusal space, and have an intact facial bone wall not requiring bone augmentation procedures. In order to be included, full mouth plaque score (FMPS) and full mouth bleeding score (FMBS) cut off values were both established at 15%. Patients were not included if they had systemic diseases that were not under control or if they had an American Society of Anesthesiologists physical status classification of III or IV. Reasons for exclusion were poor health, periodontitis, smoking, and exaggerated alcohol consumption. Cases were excluded if clinical, photographic, and radiographic data were incomplete and/or adjacent natural teeth changed after the implant treatment.

The research was conducted in private practice, and the medical devices evaluated had already been approved for clinical use. Because of the retrospective nature of this study, it was granted an exemption in writing by the local IRB. The study was conducted following the guidelines of the Declaration of Helsinki. All the patients gave their approval after they had received information about the objectives and the aims of the research.

We performed three surgical procedures, as follows:

1. Type 1 implant with SCTG
2. Type 1 implant with XCM
3. Type 1 implant without soft tissue augmentation (NG)

The selection of treatment protocol was not random but rather was made after the preoperative clinical and radiographical evaluation and discussion with the patients. Patients who agreed to undergo autogenous soft tissue graft harvest from the palate were treated as described in group 1; patients who refused to undergo a subepithelial connective tissue graft were treated using XCM as described in group 2; and patients who refused to undergo an autogenous soft tissue graft and receive XCM underwent an immediate implant placement without soft tissue augmentation (NG group). Surgeries were carried out by the same specialist in oral surgery.

**Figure 1:**
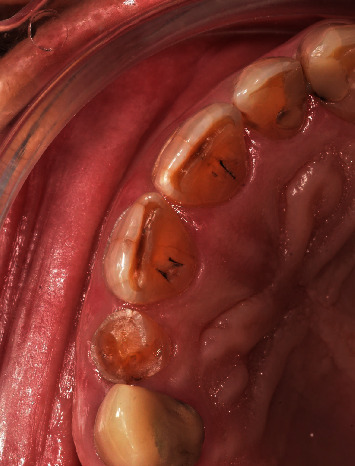
Preoperative clinical situation.

**Figure 2:**
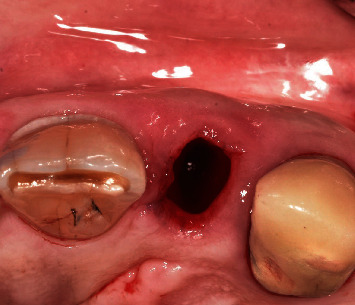
Atraumatic extraction of the fractured tooth.

**Figure 3:**
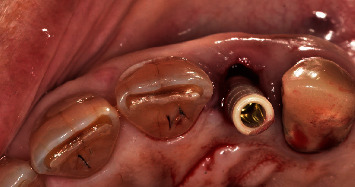
Implant placement.

**Figure 4:**
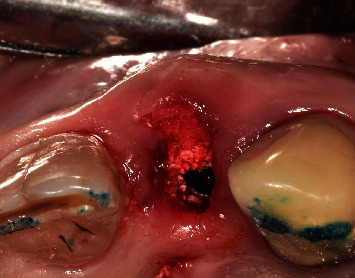
Soft tissue augmentation using the xenogenic collagen matrix.

Before initiating the extraction and implant placement, all subjects underwent periodontal procedures to establish an adequate oral hygiene condition. At least two hours before the operation, 1 g of amoxicillin was given to every participant. Antibiotics were administered for a week after the surgery. Ketoprofen 50 mg was given for pain relief at 12 hours intervals for three/four days. Patients had to use mouth rinse with chlorhexidine (0.12%) two times a day after their operation. Teeth were extracted under local anesthesia (Figure 1). A flapless approach, performed as atraumatically as possible using periotomes, was used. After using periotomes, teeth were carefully extracted by means of extraction forceps (Figure 2). Then, following tooth extraction, the surgeon carried out the debridement of the adjacent tooth surfaces and removed granulation tissue by the use of hand instruments and sterile saline rinses. The socket walls were inspected to exclude the presence of fenestration or dehiscence defects. All extraction sites had intact bone walls and received bone-level implants (Straumann Dental Implant System, MIS Implants) which were positioned in a lingual/palatal position (Figure 3) and a bone xenograft (Bio-Oss®; Geistlich Pharma AG, Wolhusen, Switzerland) was packed in the gap. The implants were placed with the shoulder located 3 mm apically from the line connecting the cementoenamel junction (CEJ) of the surrounding teeth. In all cases, primary implant stability was reached. In the SCTG group, a subepithelial connective tissue graft with a width slightly greater than the mesiodistal width of the recipient site and a thickness of 1.5 mm was taken from the palate and was positioned supraperiosteally in the buccal area using a tunnel technique. The tunnel technique was performed by raising a split flap in order to create a bilaminar envelope and avoiding an incision of the interproximal papillae. The graft was stabilized using vertical and horizontal mattress sutures (5-0 vicryl; Johnson & Johnson Gateway, Piscataway, NJ, USA). In the second group, the same augmentation procedure was performed using XCM, which was sectioned in order to obtain an adequate thickness (Figure 4) (Fibro-Gide; Geistlich Pharma AG, Wolhusen, Switzerland). In the third group, patients did not undergo surgery to increase soft tissue. In each group, the wound closure was obtained with 5-0 PTFE sutures (Omnia Srl, Fidenza, Italy). A temporary abutment was customized with flowable light-polymerizing acrylic resin and placed onto the implant. All of the implants were restored immediately with screwed temporary crown avoiding all centric and eccentric contacts with the opposing dentition. The provisional restorations had an emergence profile designed to help the growth of the soft tissues, protect the blood clot and the particles of the graft.

Sutures were removed after two weeks. After three months, the prosthetic rehabilitation was initiated, employing a digital scan generated using the CEREC AC Bluecam (Dentsply Sirona, York, PA, USA) and an implant-specific scanbody, which provided the three-dimensional registration of the implant. The abutment was torqued with 35 Ncm. Taking into consideration the screw access hole position, the permanent crown was designed to be either screw retained or cement retained using an adhesive luting composite resin (Multilink Hybrid Abutment; Ivoclar Vivadent AG, Schaan, Liechtenstein) and finally polished. All the prosthesis were completed by the same prosthodontist.

The marginal change of the peri-implant soft tissue and the evaluation of the facial soft tissue thickness (FSTT), measured as a linear change (mm) from baseline to 12 months, were assessed. Additionally, the secondary outcomes included peri-implant health status of the patient, the esthetic evaluation, and the satisfaction of the patients. All the parameters were recorded by the same trained examiner who was not involved in the clinical treatment.

All the participants underwent a cone-beam computed tomography scan before the surgery so that the bone dimensions could be evaluated preoperatively. After implant placement and at one year thereafter, intraoral radiography by the use of the long cone parallel technique was performed. A bite made of silicone (3M™ Express, 3 M ESPE Dental Products, St. Paul, USA) was placed on the holding system, allowing us to reposition it precisely during each follow-up visit. Linear measurements (mm) on the digital images were performed to record the distances of the most coronal points in the mesial and distal ridge aspects from the implant shoulder.

Patient baseline findings were considered before tooth extraction. The clinical evaluation assessed the periodontal status at the implant sites and the soft tissue thickness. An assessment of periodontal status around the implant sites was done at baseline, six months, and one year after surgery evaluating the plaque index (PI), probing depth (PD), bleeding on probing (BOP), the width of keratinized tissue (KT), and papilla index.

Marginal mucosal changes were assessed at baseline and one year following surgery by taking impressions of the implant site with a polyether impression material (Impregum™, 3M ESPE Dental Products, St. Paul, USA). The obtained casts were optically scanned using inEos ×5 (Dentsply Sirona, York, PA, USA). After this, all the files were imported for performing the measures.

The two most coronal points on the buccal (P1, P5) and lingual/palatal (P6, P10) sides, respectively, of the mesial and distal papilla were identified. Additionally, the deepest points of the buccal (P3) and lingual/palatal (P8) mucosal margins were traced. Then, P2 and P4 were identified as being 1.5 mm away from P3 on the mesial and distal aspects of the buccal mucosal margin, respectively, while P7 and P9 were identified as being 1.5 mm away from P8 on the mesial and distal aspects of the lingual/palatal mucosal margin, respectively.

The evaluation of FSTT was performed at baseline and at one year at points 2 mm from P2, P3, and P4, which were located on the mucosal margin. FSTT was classified as thick or thin preoperatively. When the tissue thickness in P3 was more than 1.5 mm, it was categorized as thick; if less than such, it was categorized as thin. The error of the method was analysed using a periodontal probe (UNC-15) after the extraction and measuring the distance of the most coronal points. Intraexaminer error technique analysis was performed.

The esthetic assessment was done with the pink esthetic score (PES). PES was calculated by assigning a score from 0 points (major compromise) to 2 points (perfect result) for 7 different variables: mesial papilla presence, distal papilla presence, soft tissue level, soft tissue contour, alveolar process deficiency, soft tissue colour, and soft tissue texture. The values to calculate PES were recorded at one year after surgery.

Patient-reported outcomes were evaluated at 12 months with a self-developed visual analogue scale (VAS) questionnaire filled out by patients, which considered their pain; overall satisfaction; and opinion about the volume, shape, and colour of the peri-implant tissue. Furthermore, the state of patients' anxiety after the informative consultation and before the beginning of the treatment was assessed. The time (in minutes) required to perform the surgery was additionally recorded.

## 3. Statistical Analysis

Values were expressed as mean and SD for continuous variables, such as linear measurements measured in mm, or absolute frequency and percentages for categorical variables. The *t*-test and analysis of variance test were used where appropriate. Simple linear regression (univariate) analysis was employed to analyze the factors independently associated with peri-implant parameters. A two-tailed value of *P* < 0.05 was considered significant. Statistical analyses were done with Stata Statistical Software, version 2014 (College Station, Texas, USA).

## 4. Results

This research involved 48 patients. Sixteen patients (mean age: 51.2 ± 13.2 years; 10 females and six males) were part of the SCTG group, 14 patients (mean age: 52.4 ± 16.6 years; eight females and six males) were included in the XCM group, and 18 patients (mean age: 47.7 ± 9.1 years; eight females and 10 males) were part of the NG group.  Table 1 shows the details of the participants.

Reasons for extraction were crown/root fractures (*n* = 32; 67%), trauma (*n* = 9; 19%), destructive carious lesions (*n* = 5; 10%), or external root resorption (*n* = 2; 4%).

A thick facial soft tissue thickness (FSTT > 1.5 mm) was observed in 27 patients, while a thin facial soft tissue thickness (FSTT ≤ 1.5 mm) was identified in 21 patients during the preoperative examination. No statistically significant difference in mean FSTT was recorded preoperatively between the three groups (*P* > 0.05).

After the surgery, all sites showed uneventful healing, with no indication of complications in the donor sites recorded. Every one of the participants had their planned treatment and all of the placed implants osseointegrated successfully, resulting in a one-year survival rate of 100%. None of the patients presented biological or technical complications, with a one-year success rate of 95%. After one year, no implants showed a radiographic marginal bone loss > 1.5 mm at the mesial and distal implant shoulders. No statistically significant correlation was observed between postoperative marginal bone loss and preoperative facial soft tissue thickness.

Furthermore, no significant differences in FSTT (*P* > 0.05) were found after treatment between the SCTG group, with a mean gain of 0.58 ± 0.45 mm, and the XCM group, with a mean gain of 0.63 ± 0.41 mm, while the NG group revealed a significant difference with a mean gain of 0.21 ± 0.30 mm (*P* < 0.05).

No significant differences were found in the buccal marginal level of points 1 and 5 between all groups after the treatment (*P* > 0.05). Patients in the NG group lost significantly more in points 2, 3, and 4 than did patients in the SCTG group or the XCM group (*P* < 0.05). In the SCTG group, the mean marginal recession of P3 was 0.34 ± 0.33. In the XCM group, the mean marginal recession of P3 was 0.39 ± 0.23. In the NG group, the mean marginal recession of P3 was 0.71 ± 0.22. No correlation was found between preoperative buccal bone thickness and postoperative changes of the buccal marginal level in all groups (points 1, 2, 3, 4, and 5). A statistically significant correlation was noted between the preoperative facial soft tissue thickness and the severity of postoperative changes that occurred in the buccal marginal level in all groups (points 2, 3, and 4). Conversely, no correlation was found between the preoperative facial soft tissue thickness and the postoperative changes of the implant papilla in all groups (points 1 and 5). No significant differences were found in the postoperative lingual/palatal marginal level for all points (*P* > 0.05). Also, no statistically significant difference was noted in the postoperative width of the keratinized mucosa between the groups (*P* > 0.05), and all patients showed a keratinized mucosa > 2 mm.

Healthy peri-implant tissues at baseline and after six and 12 months were observed in all patients. All patients displayed probing depths of less than 4 mm. Plaque scores at T12 showed that 92% (44/48) had no plaque. BOP was negative in 83% (40/48) of the participants. Only one patient of the NG group received a score of less than three points in the papilla index. No significant differences were noted between the groups for PI, BOP, and PD at one year after surgery (*P* > 0.05) (Table 2).

The visual analogue scale questionnaire revealed a significant difference only for the parameters of pain and anxiety, which were greater in the SCTG group versus the other two groups (*P* < 0.001). No significant differences were noted for the overall satisfaction and for patient opinion about volume, shape, and colour of the peri-implant mucosa at one year (*P* > 0.05) (Table 3).

A significant difference was observed in the PES, which was lower in the nongrafted site, while no difference was found between the SCTG and the XCM groups (*P* > 0.05). A statistically significant correlation was noted between the preoperative facial soft tissue thickness and the PES in the NG group. No differences were recorded in relationship to white esthetic score (WES) between all groups (*P* > 0.05) (Table 2).

A significant difference was found, however, for the time required to complete the surgery, which was higher in the SCTG group and lower for the NG group (*P* < 0.001).

## 5. Discussion

IIPP can be connected to a greater occurrence of esthetic complications because the behaviour of buccal plate remodelling is highly unpredictable [20]. Furthermore, it is hard to identify the correct vertical implant level, and surgical drills tend to move to the buccal side, thus increasing the risk of marginal recession [20].

Soft tissue augmentation techniques may be adopted to diminish the occurrence of marginal recession and improve esthetic outcomes. The biomaterial used in the XCM group was a porcine resorbable and volume-stable collagen matrix, made of reconstituted and chemically cross-linked collagen, which was indicated to improve the soft tissue volume [22, 23]. XCM represents an alternative to autogenous connective tissue grafts (SCTG), which are the gold standard in regenerative soft tissue procedures because most soft tissue substitutes have no long-term scientific evidence.

From the stand point of esthetic appearance, it may be hypothesized that placing a soft tissue graft at the same time of the immediate implant can give better results with respect to the mucosal margin level and the implant papilla [24].

In a systematic review, Lee et al. [16] found that the midbuccal mucosal level was maintained after one to two years from implant placement where soft tissue grafting was performed. In this study, it was outlined that immediate provisionalization may influence the outcomes of the research through the effects of the cervical contour and interproximal contacts [16]. Temporary restorations might guide and shape peri-implant soft tissues in the esthetic zone, reducing the marginal recession [20]. Another systematic review by Lin et al. stated that there exists a limited amount of evidence demonstrating more favourable soft tissue marginal levels by adding SCTG during the immediate implant placement [20]. These results coincide with the outcomes of three randomized controlled trials that suggested that placing SCTG leads to less recession of the midbuccal mucosa and allows us to maintain the mucosal margin at the same height as that at baseline [11, 25, 26]. Kahn et al. suggested that soft tissue augmentation caused a phenotype conversion changing the quality and quantity of the facial soft tissue, thus increasing the facial mucosal stability [9]. Our findings are in line with the aforementioned studies because a greater marginal stability in the midfacial region of the buccal gingiva was observed in both the two groups where soft tissue augmentation procedures have been performed. Furthermore, the presence of a minimum amount of peri-implant tissues is necessary because bone resorption can happen to reestablish a proper soft tissue attachment when such is not present [27]. Linkevicius et al. showed that the supracrestal soft tissue can be regarded as an important influencing factor on marginal bone stability; a specific dimension of 2.5 mm of supracrestal soft tissue may reduce marginal bone loss [28].

On the contrary, the effects of the soft tissue grafting techniques on the papilla height are controversial [16]. The present research shows no differences in the papillary region between the three groups analysed, and no correlation was found between the soft tissue phenotype and the changes in the implant papilla. This finding is probably related to the minimally invasive flap utilized to perform the graft insertion, which did not displace the papilla. Furthermore, it was demonstrated that the height of the papilla is determined by the interproximal bone level and that the use of a temporary crown can be useful in providing support to the papilla after tooth removal [9]. However in our study, most of the papilla displayed a 100% embrasure fill.

Also, the effectiveness of soft tissue graft techniques on the soft tissue thickness following IIPP is debated because the long-term changes are still unclear [16]. Lin et al. presented evidence of the ability of the connective tissue graft to modify the tissue phenotype and increase the thickness. However, FSTT preoperatively was not a risk factor for a change in the midbuccal marginal level. In a study by Rungcharassaeng et al., the mean FSTT of the grafted group was significantly higher than that of the nongrafted group, even if the palatal positioning of the implants probably helped to secure a higher FSTT in both groups after the treatment than at baseline [29]. For this reason, we believe it is crucial to increase the space between the implant restoration and soft tissue choosing not only a palatal position for the implant but also a narrow diameter as well as an abutment with a narrow contour in the deep zone. In our study, only the SCTG and XCM groups were predictable, allowing us to obtain FSTT adequate to mask the colour change induced by the restorative materials in almost all cases. According to Jung et al., zirconia did not cause a colour change in 2 mm thick soft tissue and adequate soft tissue thickness is crucial for avoiding discolouration [30].

No correlation was detected between the preoperative buccal bone thickness and the changes of the facial marginal mucosal level. However, periodontally compromised patients and smokers were excluded during the selection process and only periodontally healthy and nonsmoking patients were included, because other studies demonstrated that there is a lower marginal bone loss in these patients than in periodontally compromised and tobacco-smoking patients [31–33]. In addition, they included sites with intact bone walls, and the treatment protocol being strictly followed, which involved the palatal positioning of the implant; the use of narrow diameter implants; and the placement of a xenogenic bone graft, with a slow resorption rate between the implant and the buccal wall. Furthermore, this protocol enabled us also to limit the marginal bone loss around implants to under 1.5 mm in all the cases at one year after surgery [34]. In a study by Tarnow et al., immediate implant placement with a bone graft and a contoured healing abutment or a provisional restoration caused the least change of the ridge between all groups investigated [35, 36], and the increase in height and thickness was about 1 mm [35, 36]]. Placement of the bone graft helps to give support and volume to the hard and soft tissues, and it was shown by Araujo et al. that a xenograft material could be placed in contact with soft tissues because it becomes encapsulated in the soft tissues as a noninflammatory or benign foreign body [35, 37]. However, it is still uncertain regarding which bone grafting material could become the better solution for preserving ridge contour [35]. Furthermore, the utilization of temporary restorations seems to cause good results on maintaining soft tissue volume and helps to impart a natural appearance to the soft tissue and lessens the treatment time while adding to the comfort of the patients [38].

In our study, we found a more favourable PES in the two groups that underwent soft tissue augmentation procedures. This is in line with the results of Migliorati et al. [26] that showed a greater PES in patients undergoing SCTG, while Zuiderveld et al. reported opposing results [11]. However, all of the patients received values of PES/WES above 12 points, which is considered to be acceptable [39]. In order to achieve an esthetic restoration, the prosthetic factors also play a key role [40, 41]. The emergence profile of the abutment should be palatal to that of the adjacent teeth at the mucosal margin, and the provisional restoration should be used for shaping the peri-implant soft tissues. The emergence profile of the provisional restoration can easily be modified so that the peri-implant soft tissues can be contoured. Once the desired condition is reached, the definitive restoration can be fabricated based on the contour of the provisional restoration, thereby achieving a congruent and esthetic restorative outcome [40].

All patients revealed great levels of satisfaction with regard to the esthetic outcomes (VAS score of at least 9.0), and no differences were perceived by the patients. Finally, a reduced operative time was recorded in the patients who did not undergo soft tissue augmentation, while recurring to XCM resulted in an easier and faster solution than the subepithelial connective tissue graft.

Therapeutic alternatives to the immediate implant placement are the early implant placement and the late implant placement. Early implant placement after a soft tissue healing period of 4 to 8 weeks is a valuable treatment option which is aimed at obtaining an intact mucosa at the future implant site to allow a predictable contour augmentation on the facial aspect. This approach demonstrated successful long-term results with stable marginal bone levels and marginal soft tissue levels [41, 42]. Late implant placement is a predictable and well-documented procedure with successful results which requires a healing period post extraction of 6 months or longer. For this reason, this is the least attractive option for the patient due to the long treatment period [43, 44].

This study had limitations, including the short-term follow-up, the small patient sample, the allocation bias of the patients, and the retrospective nature of the study. Furthermore, there is a lack in literature of studies with a longer observation period which is required to confirm the stability of the grafted areas [26]. Long-term prospective clinical investigations should be carried out to elucidate the clinical and biological reliability of soft tissue grafting procedures performed as well as of the prosthetic approaches used over the years.

## 6. Conclusion

The favourable success rate recorded in all groups confirmed immediate implant placement as a choice of treatment for replacing anterior single tooth. In soft tissue augmentation, XCM constituted a viable alternative to SCTG in terms of facial soft tissue thickness, buccal marginal stability, and PES.

## Figures and Tables

**Table 1:** Patient demographics and clinical features.

	SCTG	XCM	NG
Female	10	8	8
Male	6	6	10
Age	51.2 ± 13.2	52.4 ± 16.6	47.7 ± 9.1
Incisor	7	6	9
Canine	2	0	1
First premolar	7	8	8
Diameter 3.3	10	8	13
Diameter 4.1	5	6	5
Diameter 4.8	1	0	0

**Table 2:** Implant clinical and radiographical outcomes.

	SCTG	XCM	NG
Radiographic marginal bone loss (MBL) mesial	0.3 ± 0.1	0.3 ± 0.1	0.3 ± 0.1
Radiographic marginal bone loss (MBL) distal	0.3 ± 0.1	0.3 ± 0.1	0.4 ± 0.1
Pink esthetic score/white esthetic score (PES/WES)	18.6 ± 1.3	18.4 ± 1.7	17.0 ± 1.9
Probing depth (PD) > 4 mm	16	14	18
Bleeding on probing (BoP) patients	3/16	2/14	3/18
Bleeding on probing (BoP) sites	5/96	4/84	6/108
Papilla index < 3	0	0	0

**Table 3:** Patient satisfaction regarding anxiety, overall satisfaction, and esthetics.

	SCTG	XCM	NG
Q1: How high was your anxiety after the informative consultation and before the beginning of the treatment?^1^	6.5 ± 1.3	4.5 ± 2	4 ± 1.7
Q2: Did you experience pain after the procedure?^2^	6.7 ± 0.9	3.6 ± 2.3	2.8 ± 2
Q3: Are you satisfied with the prosthetic rehabilitation?^2^	9 ± 0.8	8.8 ± 0.8	8.7 ± 0.6
Q4: What is your opinion on the gingival volume?^2^	9.1 ± 0.8	8.8 ± 0.8	8.7 ± 0.6
Q5: What is your opinion on the gingival shape?^2^	9 ± 0.7	9.1 ± 0.8	9 ± 0.7
Q6: What is your opinion on the gingival colour?^2^	8.5 ± 0.8	8.6 ± 0.8	8.6 ± 0.7

^1^VAS: no anxiety 0–10 top level of anxiety; ^2^VAS: unsatisfactory 0–10 excellent.

## Data Availability

The data of the manuscript are available from the authors on request. Prof. Giulio Gasparini should be contacted to receive them.

## References

[1] Chen S. T., Wilson T. G., Hämmerle C. H. (2004). Immediate or early placement of implants following tooth extraction: review of biologic basis, clinical procedures, and outcomes. *The International Journal of Oral & Maxillofacial Implants*.

[2] Cabello G., Rioboo M., Fábrega J. G. (2013). Immediate placement and restoration of implants in the aesthetic zone with a trimodal approach: soft tissue alterations and its relation to gingival biotype. *Clinical Oral Implants Research*.

[3] Moro A., De Angelis P., Pelo S. (2018). Alveolar ridge augmentation with maxillary sinus elevation and split crest: comparison of 2 surgical procedures. *Medicine (Baltimore)*.

[4] van Nimwegen W. G., Raghoebar G. M., Zuiderveld E. G., Jung R. E., Meijer H. J. A., Mühlemann S. (2018). Immediate placement and provisionalization of implants in the aesthetic zone with or without a connective tissue graft: a 1-year randomized controlled trial and volumetric study. *Clinical Oral Implants Research*.

[5] Farronato D., Mangano F., Briguglio F., Iorio-Siciliano V., Riccitiello F., Guarnieri R. (2014). Influence of Laser-Lok surface on immediate functional loading of implants in single-tooth replacement: a 2-year prospective clinical study. *The International Journal of Periodontics & Restorative Dentistry*.

[6] Guarnieri R., Placella R., Testarelli L., Iorio-Siciliano V., Grande M. (2014). Clinical, radiographic, and esthetic evaluation of immediately loaded laser microtextured implants placed into fresh extraction sockets in the anterior maxilla: a 2-year retrospective multicentric study. *Implant Dentistry*.

[7] Del Amo F. S. L., Yu S.-H., Sammartino G. (2020). Peri-implant soft tissue management: Cairo Opinion Consensus Conference. *International Journal of Environmental Research and Public Health*.

[8] Guarnieri R., Grande M., Ippoliti S., Iorio-Siciliano V., Riccitiello F., Farronato D. (2015). Influence of a Laser-Lok surface on immediate functional loading of implants in single-tooth replacement: three-year results of a prospective randomized clinical study on soft tissue response and esthetics. *The International Journal of Periodontics & Restorative Dentistry*.

[9] Kan J. Y. K., Rungcharassaeng K., Morimoto T., Lozada J. (2009). Facial gingival tissue stability after connective tissue graft with single immediate tooth replacement in the esthetic zone: consecutive case report. *Journal of Oral and Maxillofacial Surgery*.

[10] Chen S. T., Buser D. (2014). Esthetic outcomes following immediate and early implant placement in the anterior maxilla—a systematic review. *Int J Oral Maxillofac Implants*.

[11] Zuiderveld E. G., Meijer H. J. A., den Hartog L., Vissink A., Raghoebar G. M. (2018). Effect of connective tissue grafting on peri-implant tissue in single immediate implant sites: a RCT. *Journal of Clinical Periodontology*.

[12] Araújo M. G., Lindhe J. (2005). Dimensional ridge alterations following tooth extraction. An experimental study in the dog. *Journal of Clinical Periodontology*.

[13] Fickl S., Zuhr O., Wachtel H., Bolz W., Huerzeler M. (2008). Tissue alterations after tooth extraction with and without surgical trauma: a volumetric study in the beagle dog. *Journal of Clinical Periodontology*.

[14] Grunder U. (2011). Crestal ridge width changes when placing implants at the time of tooth extraction with and without soft tissue augmentation after a healing period of 6 months: report of 24 consecutive cases. *The International Journal of Periodontics & Restorative Dentistry*.

[15] Iorio-Siciliano V., Blasi A., Sammartino G., Salvi G. E., Sculean A. (2020). Soft tissue stability related to mucosal recession at dental implants: a systematic review. *Quintessence International*.

[16] Lee C. T., Tao C. Y., Stoupel J. (2016). The effect of subepithelial connective tissue graft placement on esthetic outcomes after immediate implant placement: systematic review. *Journal of Periodontology*.

[17] Guarnieri R., Serra M., Bava L., Grande M., Farronato D., Iorio-Siciliano V. (2014). The impact of a laser-microtextured collar on crestal bone level and clinical parameters under various placement and loading protocols. *The International Journal of Oral & Maxillofacial Implants*.

[18] Huber S., Zeltner M., Hämmerle C. H. F., Jung R. E., Thoma D. S. (2018). Non-interventional 1-year follow-up study of peri-implant soft tissues following previous soft tissue augmentation and crown insertion in single-tooth gaps. *Journal of Clinical Periodontology*.

[19] Iorio-Siciliano V., Marzo G., Blasi A., Cafiero C., Mignogna M., Nicolò M. (2014). Soft and hard tissue modifications at immediate transmucosal implants (with Laser-Lok microtextured collar) placed into fresh extraction sites: a 6-month prospective study with surgical reentry. *International Journal of Periodontics & Restorative Dentistry*.

[20] Lin G. H., Chan H. L., Wang H. L. (2014). Effects of currently available surgical and restorative interventions on reducing midfacial mucosal recession of immediately placed single-tooth implants: a systematic review. *Journal of Periodontology*.

[21] Testori T., Weinstein T., Scutellà F., Wang H. L., Zucchelli G. (2018). Implant placement in the esthetic area: criteria for positioning single and multiple implants. *Periodontol 2000*.

[22] Thoma D. S., Zeltner M., Hilbe M., Hämmerle C. H., Hüsler J., Jung R. E. (2016). Randomized controlled clinical study evaluating effectiveness and safety of a volume-stable collagen matrix compared to autogenous connective tissue grafts for soft tissue augmentation at implant sites. *Journal of Clinical Periodontology*.

[23] Zeltner M., Jung R. E., Hämmerle C. H. F., Hüsler J., Thoma D. S. (2017). Randomized controlled clinical study comparing a volume-stable collagen matrix to autogenous connective tissue grafts for soft tissue augmentation at implant sites: linear volumetric soft tissue changes up to 3 months. *Journal of Clinical Periodontology*.

[24] Thoma D. S., Buranawat B., Hämmerle C. H., Held U., Jung R. E. (2014). Efficacy of soft tissue augmentation around dental implants and in partially edentulous areas: a systematic review. *Journal of Clinical Periodontology*.

[25] Yoshino S., Kan J. Y., Rungcharassaeng K., Roe P., Lozada J. L. (2014). Effects of connective tissue grafting on the facial gingival level following single immediate implant placement and provisionalization in the esthetic zone: a 1-year randomized controlled prospective study. *The International Journal of Oral & Maxillofacial Implants*.

[26] Migliorati M., Amorfini L., Signori A., Biavati A. S., Benedicenti S. (2015). Clinical and aesthetic outcome with post-extractive implants with or without soft tissue augmentation: a 2-year randomized clinical trial. *Clinical Implant Dentistry and Related Research*.

[27] Berglundh T., Lindhe J. (1996). Dimension of the periimplant mucosa. Biological width revisited. *Journal of Clinical Periodontology*.

[28] Linkevicius T., Apse P., Grybauskas S., Puisys A. (2009). The influence of soft tissue thickness on crestal bone changes around implants: a 1-year prospective controlled clinical trial. *The International Journal of Oral & Maxillofacial Implants*.

[29] Rungcharassaeng K., Kan J. Y., Yoshino S., Morimoto T., Zimmerman G. (2012). Immediate implant placement and provisionalization with and without a connective tissue graft: an analysis of facial gingival tissue thickness. *The International Journal of Periodontics & Restorative Dentistry*.

[30] Jung R. E., Sailer I., Hämmerle C. H., Attin T., Schmidlin P. (2007). In vitro color changes of soft tissues caused by restorative materials. *The International Journal of Periodontics & Restorative Dentistry*.

[31] Rasperini G., Siciliano V. I., Cafiero C., Salvi G. E., Blasi A., Aglietta M. (2014). Crestal bone changes at teeth and implants in periodontally healthy and periodontally compromised patients. A 10-year comparative case-series study. *Journal of Periodontology*.

[32] Aglietta M., Siciliano V. I., Rasperini G., Cafiero C., Lang N. P., Salvi G. E. (2011). A 10-year retrospective analysis of marginal bone-level changes around implants in periodontally healthy and periodontally compromised tobacco smokers. *Clinical Oral Implants Research*.

[33] Matarasso S., Rasperini G., Iorio Siciliano V., Salvi G. E., Lang N. P., Aglietta M. (2010). A 10-year retrospective analysis of radiographic bone-level changes of implants supporting single-unit crowns in periodontally compromised vs. periodontally healthy patients. *Clinical Oral Implants Research*.

[34] Papaspyridakos P., Chen C. J., Singh M., Weber H. P., Gallucci G. O. (2011). Success criteria in implant dentistry: a systematic review. *Journal of Dental Research*.

[35] Tarnow D. P., Chu S. J., Salama M. A. (2014). Flapless postextraction socket implant placement in the esthetic zone: part 1. The effect of bone grafting and/or provisional restoration on facial-palatal ridge dimensional change—a retrospective cohort study. *International Journal of Periodontics & Restorative Dentistry*.

[36] Chu S. J., Salama M. A., Garber D. A. (2015). Flapless postextraction socket implant placement, part 2: the effects of bone grafting and provisional restoration on peri-implant soft tissue height and thickness—a retrospective study. *Int J Periodontics Restorative Dent*.

[37] Araújo M. G., Linder E., Lindhe J. (2011). Bio-Oss collagen in the buccal gap at immediate implants: a 6-month study in the dog. *Clinical Oral Implants Research*.

[38] Chu S. J., Saito H., Salama M. A. (2018). Flapless postextraction socket implant placement, part 3: the effects of bone grafting and provisional restoration on soft tissue color change—a retrospective pilot study. *The International Journal of Periodontics & Restorative Dentistry*.

[39] Jones A. R., Martin W. (2014). Comparing pink and white esthetic scores to layperson perception in the single-tooth implant patient. *Int J Oral Maxillofac Implants*.

[40] Steigmann M., Monje A., Chan H. L., Wang H. L. (2014). Emergence profile design based on implant position in the esthetic zone. *International Journal of Periodontics & Restorative Dentistry*.

[41] De Angelis P., Passarelli P. C., Gasparini G., Boniello R., D'Amato G., De Angelis S. (2020). Monolithic CAD-CAM lithium disilicate versus monolithic CAD-CAM zirconia for single implant-supported posterior crowns using a digital workflow: a 3-year cross-sectional retrospective study. *The Journal of Prosthetic Dentistry*.

[42] Buser D., Wittneben J., Bornstein M. M., Grütter L., Chappuis V., Belser U. C. (2011). Stability of contour augmentation and esthetic outcomes of implant-supported single crowns in the esthetic zone: 3-year results of a prospective study with early implant placement postextraction. *Journal of Periodontology*.

[43] Buser D., Chappuis V., Belser U. C., Chen S. (2017). Implant placement post extraction in esthetic single tooth sites: when immediate, when early, when late?. *Periodontol 2000*.

[44] De Angelis P., De Angelis S., Passarelli P. C., Liguori M. G., Manicone P. F., D'Addona A. (2019). Hard and soft tissue evaluation of different socket preservation procedures using leukocyte and platelet-rich fibrin: a retrospective clinical and volumetric analysis. *Journal of Oral and Maxillofacial Surgery*.

